# Granulocyte Colony-Stimulating Factor in the Treatment of Acute Radiation Syndrome: A Concise Review

**DOI:** 10.3390/molecules19044770

**Published:** 2014-04-16

**Authors:** Michal Hofer, Milan Pospíšil, Denisa Komůrková, Zuzana Hoferová

**Affiliations:** Department of Molecular Cytology and Cytometry, Institute of Biophysics, v.v.i., Academy of Sciences of the Czech Republic, Královopolská 135, Brno CZ-612 65, Czech Republic; E-Mails: jirina.pospisil@seznam.cz (M.P.); komurkova@ibp.cz (D.K.); hoferovaz@centrum.cz (Z.H.)

**Keywords:** granulocyte colony-stimulating factor, radiation accidents, acute radiation syndrome, radiation-induced myelosuppression

## Abstract

This article concisely summarizes data on the action of one of the principal and best known growth factors, the granulocyte colony-stimulating factor (G-CSF), in a mammalian organism exposed to radiation doses inducing acute radiation syndrome. Highlighted are the topics of its real or anticipated use in radiation accident victims, the timing of its administration, the possibilities of combining G-CSF with other drugs, the ability of other agents to stimulate endogenous G-CSF production, as well as of the capability of this growth factor to ameliorate not only the bone marrow radiation syndrome but also the gastrointestinal radiation syndrome. G-CSF is one of the pivotal drugs in the treatment of radiation accident victims and its employment in this indication can be expected to remain or even grow in the future.

## 1. Introduction

The treatment of radiation accident victims has been in the focus of attention of investigators in many laboratories. Radiation accidents are fortunately infrequent but their health consequences are serious and they have the potential of resulting in large-scale events [1]. Working groups on the management of acute radiation syndrome have been established [2,3]. The research area of Therapeutic Agents (Postexposure Treatment) has been given top priority on the priority list of research areas for radiological nuclear threat countermeasures [4].

## 2. Brief Introductory Statements Concerning Granulocyte Colony-Stimulating Factor (G-CSF) and Mechanisms of Its Action

Granulocyte colony-stimulating factor (G-CSF, molecular weight about 20,000) is a glycoprotein which regulates especially the production of neutrophils; it is produced by activated macrophages, endothelial cells and fibroblasts; using recombinant DNA technology, G-CSF can be produced in large quantity in *Escherichia coli* and mammalian cells, and it is widely used clinically to treat patients suffering from neutropenia after cancer chemotherapy [5]. Besides the stimulatory action of G-CSF on proliferation of the hematopoietic cells, the antiapoptotic action of G-CSF in the radiation-induced myelosuppression is emphasized [6]. Also the recently reported G-CSF-induced maturation of monocytes/macrophages [7] can be relevant to the role of this growth factor in acute radiation syndrome. Aside from its efficacy in the hematopoietic system, G-CSF also influences functioning of other systems in the mammalian organism like the cardiovascular [8] or neural ones [9].

## 3. A Brief Overview of Key Stages in the Use of Granulocyte Colony-Stimulating Factor (G-CSF) for the Treatment of Acute Radiation Syndrome

Since the first reports on hematopoiesis-stimulating effects of granulocyte colony-stimulating factor (G-CSF) in the early eighties (e.g., [10]), more than 14,000 articles have been published on the topic of features and actions of this substance in animal experiments and, subsequently, also in the clinical practice. At the beginning of the nineties, reports appeared both on the ability of G-CSF to positively influence hematopoiesis and survival in lethally irradiated mice [11] and on the reversal of radiation-induced neutropenia in man [12]. Though yet another growth factor, namely the granulocyte-macrophage colony-stimulating factor (GM-CSF), had been therapeutically administered to victims of some radiation accidents (Goiânia, Brazil, 1987 [13]; Henan Province, China, 1987 [14]), radiation-exposed patients from several subsequent accidents were treated with G-CSF (Tokai-mura, Japan, 1999 [15]; Dakar, Senegal, 2006 [3], and Fleurus, Belgium, 2006 [3]). Due to its efficacy, G-CSF has obtained the Emergency Use Investigational New Drug status for the acute radiation syndrome from the United States Food and Drug Administration (FDA) [16], and is currently available in radiation stockpiles that have been developed in the U.S. and by the World Health Organization [17].

In spite of undeniable positive effects of G-CSF when administered to radiation victims, as well as to patients with neutropenia induced by other myelosuppressive factors [18], extensive investigations of this growth factor have continued. High numbers of publications on G-CSF and its effects in irradiated animals and humans do not enable us to list and to discuss them in full. However, there exist some areas of problems whose studies might influence future clinical procedures aimed at the therapy of acute radiation syndrome with G-CSF. Selected problem areas are briefly discussed in the following paragraphs.

## 4. Timing of G-CSF after Irradiation

Studies evaluating the efficacy of therapeutically (post-irradiation) administered G-CSF applied mostly the administration schedules of protracted repeated dosing in the course of the first 10 to 20 days after irradiation. This approach was used in experiments on mice (e.g., [19]), dogs (e.g., [20]), nonhuman primates (e.g., [21]), or minipigs (e.g., [7]). As regards the medical use of G-CSF, there exists a recommendation based on the above-mentioned principle of protracted post-irradiation G-CSF administration formulated as “First clinical consensus for evidence-based management of the hematopoietic syndrome resulting from exposure to ionizing radiation” [22].

However, more than twenty years ago a finding suggesting a significant survival enhancement in lethally irradiated mice administered a single dose of G-CSF early after irradiation was presented; the efficacy of G-CSF administration was the better the earlier after irradiation G-CSF was given, with the interval of 2 h post-irradiation being the optimum one [23]. The early post-irradiation effects of G-CSF and other growth factors were experimentally verified especially by Hérodin and coworkers [24,25,26], who interpreted them as a result of their antiapoptotic action and formulated the “the sooner the better” principle of post-irradiation administration of growth factors [6].

Taking into account the two principles of therapy of the acute radiation syndrome with G-CSF, it can be postulated that both the approaches of early and protracted administration of G-CSF are useful, the first one struggling with the acute radiation-induced apoptosis, the second one supporting the regeneration processes in the hematopoietic tissue.

## 5. Therapy using Combinations of G-CSF with Other Agents

The topic of modifying radiation damage using a combined agent treatment regimen was thoroughly discussed by Weiss and coworkers already in 1990 [27]. The expected benefits of the combined therapy of acute radiation syndrome are: (a) enhancement of efficacy of the treatment if the combined agents influence the same function; (b) extension of the spectrum of effectiveness of the treatment if the combined agents influence different functions; and (c) reduction of toxicity of the treatment if combining the agents enables us to reduce the doses of the individual agents. The possibilities of combining G-CSF with other agents are wide. Examples of such combinations are given below. In addition to the treatment with specific hematopoiesis-modulating drugs, the therapy spectrum of radiation victims also includes administration of antimicrobial agents and supportive care (antiemetics, fluids, electrolytes, antidiarrheal agents, analgesic agents, and others) [2].

One of the possibilities of improving the results of the treatment of acute radiation syndrome is to combine G-CSF with other cytokines. The experiments of MacVittie and coworkers from the nineties [28] may serve as an example. When combining G-CSF with a synthetic interleukin-3 receptor agonist, a positive effect on recovery from both neutropenia and thrombocytopenia was observed. Another example of combining cytokines in the experimental treatment of acute radiation syndrome is the antiapoptotic cytokine combinations of Hérodin and coworkers, comprising the stem cell factor, Flt-3 ligand, thrombopoietin, interleukin-3, and G-CSF [6,24,25,26]. The combination of G-CSF and GM-CSF in the treatment of humans with the acute radiation disease should be mentioned in connection with the U.S. Radiation Accident Registry maintained by the Radiation Emergency Assistance Center/Training Site (REAC/TS), where 28 patients treated with G-CSF and GM-CSF (in most instances concurrently) were listed; 25 out of these 28 patients appeared to have stimulated neutrophil recovery [2].

G-CSF can also be combined with the classical radioprotector WR-2721 (see, e.g., [29,30]). However, this approach is limited to situations in which exposure to radiation is to be expected since WR-2721 is effective only when administered before irradiation, as a radioprotector. The combination of G-CSF + WR-2721 is, thus, a combination of radiation protection (WR-2721) with the therapy of the subsequently incurred radiation syndrome (G-CSF).

Adenosine receptor agonists belong to substances that have been shown to stimulate hematopoiesis in irradiated mice (for review, see [31]). The hematopoiesis-stimulating effects of activation of adenosine receptors in radiation-exposed mice, achieved by a combination of adenosine monophosphate and dipyridamole, were found to be potentiated by concomitant administration of G-CSF [32,33]. Similar findings were obtained when the drugs activating adenosine receptors and G-CSF were administered to mice in which a severe myelosuppression was induced by a combination of ionizing radiation and a cytotoxic drug, 5-fluorouracil [34]. G-CSF was also observed to potentiate hematopoiesis-stimulating effects of IB-MECA, a selective agonist of adenosine A_3_ receptors, in mice with radiation-induced myelosuppression [35]. The above-mentioned examples clearly illustrate the appropriateness of combining G-CSF with other agents in attempts to ameliorate the progress of acute radiation disease.

## 6. Utilization of G-CSF in Nonhuman Primate Model of Acute Radiation Disease

The usefulness of G-CSF in the treatment of acute radiation disease in the nonhuman primate model was already demonstrated in earlier studies (e.g., [21,28,36]). In these studies, G-CSF or its pegylated form, pegfilgrastim, was used. Testing of pegfilgrastim was justified by its much longer half-time in humans (e.g., [18]). Recently refined nonhuman primate model of the hematopoietic radiation syndrome plus medical management [37] has been utilized for testing the effects of G-CSF on survival of lethally irradiated rhesus macaques [38], as well as for evaluation of the action of pegfilgrastim on neutrophil recovery in potentially lethally irradiated rhesus macaques [39]. In both the studies, significant therapeutic achievements have been obtained, namely even following abbreviated administration schedules.

## 7. Pharmacological Induction of Endogenous G-CSF Production

G-CSF in an irradiated organism can act either as a consequence of its therapeutical administration or on the basis of its increased production induced by other drugs, which themselves were tested for their ability to ameliorate the acute radiation damage to the mammalian organism. This “indirect effect” was described, e.g., for vitamin E analogs, like tocopherol succinate [40] and γ-tocotrienol [41], genistein, a phytochemical [42], CBLB502, a derivative of the *Salmonella* fagellin [43], 5-androstenediol, an adrenocortical steroid hormone [44], meloxicam, a cyclooxygenase-2 inhibitor [45,46,47], IB-MECA, an adenosine A_3_ receptor agonist [48], and glucan, an immunomodulator [49]. 

It follows from these reports that the acute radiation syndrome-mitigating effects of G-CSF can also be employed by the induction of its endogenous production by other agents. It is tempting to speculate that some cheap and readily available drug, like e.g., meloxicam, could play a role in the first aid treatment of the victims of a contingent massive radiation accident.

## 8. Action of G-CSF on the Gastrointestinal Radiation Syndrome

The primary curative effects of G-CSF are those stimulating the regeneration of the hematopoietic tissues and, as such, they are utilized in connection with acute radiation syndrome preferentially after radiation doses inducing the bone marrow syndrome of the acute radiation disease. However, a high-dose radiation exposure also causes a severe intestinal damage (gastrointestinal syndrome of the acute radiation disease), which then plays a pivotal role in patient survival (e.g., [15]). In 2008, a report appeared suggesting that G-CSF also possesses important functions in the gastrointestinal tissues (e.g., [50]). Contingent abilities of G-CSF to ameliorate the intestinal radiation damage were recently successfully tested by Kim and coworkers [51,52]. The authors showed that G-CSF reduced the levels of proinflammatory cytokines interleukin-6 and tumor necrosis factor-α and protected intestinal mucosal injury through its anti-apoptotic and anti-inflammatory effects.

It can be inferred from these observations that G-CSF can be applied with benefit in the treatment of acute radiation syndrome to patients exposed to a wide spectrum of radiation doses comprising those which induce the bone marrow and gastrointestinal radiation syndromes.

## 9. Conclusions

Although efforts aimed at developing medically effective radiation countermeasure approaches, including radiation protectors, mitigators, and therapeutics, were initiated more than half a century ago, no safe and effective radiation countermeasure has been approved by FDA for the acute radiation syndrome by now [16]. Granulocyte colony-stimulating factor (G-CSF) plays a pivotal role in the treatment of victims of radiation accidents. G-CSF is effective when administered either singly early after irradiation or when applied in a protracted several-day treatment regimen. This growth factor can be successfully combined with other growth factors or other drugs to further improve the outlook of the therapy. Various drugs can induce the endogenous production of G-CSF and, thus, mediate its effects. It can be concluded that, in the treatment of the acute radiation disease, G-CSF has a rich history of its experimental testing and has found practical application in clinical medicine.

As a result of the achievements obtained in the course of the investigations on the topic of using G-CSF in the treatment of the acute radiation syndrome, G-CSF has obtained the Emergency Use Investigational New Drug status for the acute radiation syndrome from FDA [16], as stated above. Nevertheless, there still exist topics deserving further studies on this growth factor, and it can be expected that the important role of G-CSF in treating radiation accident victims will persist and grow also in the future.
